# Editorial: Gut microbiota dynamics and nutritional strategies in porcine weaning period

**DOI:** 10.3389/fcimb.2025.1637276

**Published:** 2025-07-17

**Authors:** Yang Li, Shuzhen Jiang

**Affiliations:** Key Laboratory of Efficient Utilization of Non-Grain Feed Resources, Ministry of Agriculture and Rural Affairs, College of Animal Science and Technology, Shandong Agricultural University, Tai’an, China

**Keywords:** gut microbiota, nutrition intervention, diarrhea prevention, weaning, piglets

Weaning piglets from their sow is one of the most stressful events in the pig’s life. This transition triggers a huge stress response because of multiple factors such as environmental factors, maternal separation, and infection, leading to dramatic alterations of gut microbiota (Tang et al., 2022). Gut microbiota plays a vital role in host health by providing nutrients, modulating gastrointestinal development, shaping the immune system and competitive inhibition of pathogens. Disorder of gut microbiota will destroy the dynamic environment in intestinal mucosa where the host constantly interacts with trillions of commensal microorganisms, resulting in intestinal barrier dysfunction, oxidative stress, and inflammatory response of the host (Gresse et al., 2017). Nutritional interventions via changing dietary patterns are effective strategies for the modulation of gut microbiota. Therefore, studies that focus on the role of nutritional interventions in the modulation of gut microbiota during the porcine gestating and weaning period and further molecular mechanisms by targeting related signaling pathways, as well as the interactions between these pathways and gut microbiota *in vitro* and *in vivo*, are urgently needed, which can help us to comprehensively understand the meaning of the application of nutritional strategies in the pig production industry. Accordingly, we have introduced the Research Topic entitled “Gut Microbiota Dynamics and Nutritional Strategies in Porcine Weaning Period”, focusing on intestinal microorganisms and nutritional physiology of weaning piglets. The articles submitted to the Research Topic include four research articles and a review, covering multiple nutritional strategies, including the exploration of bacterial regulatory mechanisms, replacement of traditional raw materials and trace elements, as well as the application of novel feed sources. Below is the summary of these studies.

In response to the growing concerns of environmental sustainability and public health, the use of antibiotic and zinc oxide is gradually restricted. However, effective and widely applicable alternatives are still lacking. A better understanding of the mechanisms of how antibiotic and zinc oxide function can be beneficial for the development and optimization of substitute products. Ortiz Sanjuán et al. conducted experiments across multiple porcine farms to evaluate the impact of these compounds under practical production conditions. They compared the relationship between weaning piglets and the use of antibiotic and zinc oxide, considering both post-weaning days and their living environment. Their findings showed that the changes of intestinal microbiota were more related with the days post weaning instead of the living environment. The treatment of antibiotic and zinc oxide could change the gut bacterial composition. Specifically, they pointed out that although the treated piglets experienced diarrhea in the early days after weaning, their gut microbiota composition was similar to that of the older, healthy piglets, which exhibited the important function of antibiotic and zinc oxide in molding and stabilizing intestine microbiota during the weaning period. This study provides valuable insights into weaning stress, gut microbial balance and the roles of antibiotics and zinc oxide in diarrhea prevention.

From a novel and close to nature perspective, Yao et al. investigated whether providing grass hay, which are commonly chewed by wild piglets, could help mitigated the adverse effects induced by weaning stress in suckling piglets. Grass hay, which was provided in addition to conventional creep feed, had positive effects on the intestinal development on suckling piglets, including improving the intestinal morphology and promoting the production of short-chain fat acids in proximal sections of large intestine. Although grass hay is rich in fiber, which theoretically could alter gut microbiota composition, it had only minimal effects on the colonic bacterial community (Shang et al., 2021). All in all, this work presents a new approach that integrates ecological behavior with nutrition strategies, and offered a new feasible direction for nutrient intervention during weaning period in piglets.

Iron is the inevitable trace elements nutrition in animals. Fu et al. evaluated the effects of iron hydroxy methionine analog chelate (Fe-HMA), a novel organic iron feed additive, compared with the traditional inorganic iron source in weaned piglets. The findings revealed that Fe-HMA performed better than the ferrous sulfate in promoting growth performance and improving serum antioxidant status. Notably, Fe-HMA showed complex effects on the intestinal bacteria. On one hand, it increased the relative abundance of the microbiota (including *Gemmiger*, *Subdoligranulum*, and *Dorea*) with the function on ameliorating intestinal inflammation. On the other hand, some pathogen abundance was increased, which, however, showed no significantly adverse effects on the physiology. This research provided a theory basis for the use of organic iron sources in nutritional strategies in weaned piglets.

Protein resource shortages in animal feed are a common issue in plenty of countries. Yeast-derived products are diverse, and its potential still need to be explored. The gut microbiota of piglets is influenced by the microbiota of the sow. Zhou et al. innovatively employed yeast protein, a type of yeast-derived product, as a substitute for fishmeal, the traditional high-quality protein resource. They evaluated its effects on sows with different genetic background in late gestion and lactation. Except for protein replacement, yeast protein exhibited beneficial effects on sows’ health. It reduced the backfat loss in late lactation, thereby supporting energy reserves and preparing for the subsequent reproductive cycle. Meanwhile, it enhanced immune function and reduced pro-inflammation cytokines levels in late lactation. As a dietary protein source, yeast protein also modulated the gut bacteria structure in sows by enriching the beneficial microbes and suppressing the colonization of harmful bacteria. This study provides valuable insights and theoretical support for the application of yeast protein in sow nutrition and management.

Short-chain fatty acids (SCFAs), as the main end-products produced by intestinal microorganisms fermenting dietary fiber, play an important role in maintaining intestinal health (Wang et al., 2019). Liu et al. systematically reviewed the impacts of SCFAs in regulating intestinal microbiota structure, immune function, inflammatory response and intestinal barrier in piglets. The review highlights that different SCFA types exert different physiological roles, for example, butyrate acid provides energy for intestinal epithelial cells, while propionate acid promotes the growth and development of intestinal villi. SCFAs can also regulate the intestinal microbiota through feedback mechanisms by promoting the colonization of beneficial bacteria such as *Lactobacillus* and *Bifidobacterium*, and inhibiting harmful bacteria such as *Salmonella* and *Escherichia coli*. Dietary SCFAs derivatives supplementation play a similar regulatory role as SCFAs. Moreover, SCFAs can enhance multiple kinds of immune functions and effectively alleviate intestinal inflammation in piglets. This review provides a theoretical basis and direction for the application of short-chain fatty acids in pig production and intestinal disease intervention.

The articles in this Research Topic offer new insights into nutritional regulation strategies for weaned piglets from multiple perspectives. Collectively, they enhance our understanding of the relationship between the porcine nutrition physiology and gut microbiota during weaning period. These articles provide theoretical support and practical possibilities for modulating intestinal microbiota to promote piglets’ health through nutrition interventions, especially under the context of restrictions on antibiotic and zinc oxide in animal feed.
